# Verbal creativity in autism: comprehension and generation of metaphoric language in high-functioning autism spectrum disorder and typical development

**DOI:** 10.3389/fnhum.2014.00615

**Published:** 2014-08-11

**Authors:** Anat Kasirer, Nira Mashal

**Affiliations:** ^1^School of Education Bar-Ilan UniversityRamat-Gan, Israel; ^2^Gonda Multidisciplinary Brain Research Center, Bar-Ilan UniversityRamat-Gan, Israel

**Keywords:** autism, novel metaphors, executive functioning, metaphor generation

## Abstract

Studies on creativity in participants with autism generally show impoverished performance as well as deficient comprehension of metaphoric language. However, very little is known about the ability to generate metaphors in this population. The present study examines verbal creativity in adults with autism-spectrum disorder (ASD) through tasks that rely on novel metaphoric language. Seventeen adults with ASD (mean age = 21.06) and 17 typically developing peers (mean age = 22.71) participated in the study. A multiple-choice questionnaire consisting of conventional and novel metaphors was used to test comprehension, and a sentence completion questionnaire was used to test generation of creative language. Results show similar performance in comprehension of conventional and novel metaphors in both groups, whereas adults with ASD generated more creative metaphors relative to the control group. Scores on tests of vocabulary and naming contributed to the prediction of conventional metaphor comprehension, while scores on tests of mental flexibility contributed to the prediction of novel metaphor comprehension. In addition, scores on a test of non-verbal intelligence contributed to the prediction of metaphor generation. The study points to unique verbal creativity in ASD.

## INTRODUCTION

People with autistic-spectrum disorder (ASD) experience difficulties in comprehension of figurative language, and tend to give literal interpretation to ambiguous syntactic expressions, to phrases that convey irony, humor, or sarcasm, to idioms, or to metaphors (Happé, 1993, 1995; Kerbel and Grunwell, 1998; Rapin and Dunn, 2003; Adachi et al., 2004; Rundblad and Annaz, 2010; Mashal and Kasirer, 2011). Despite extensive research on comprehension of metaphoric language in autism, very little is known about how people with ASD generate metaphors. In fact, earlier research on creativity in ASD has focused primarily on non-verbal rather than on verbal abilities. Findings have mostly documented deficient performance in ASD, especially when using tasks that are imagination-oriented (e.g., Frith, 1972; Lewis and Boucher, 1991; Craig and Baron-Cohen, 1999). The present study investigates comprehension and generation of novel metaphors in ASD.

In a recent study we found that children with ASD (aged 12–15) differ from typically developing (TD) children in comprehension of conventional metaphors but not in comprehension of novel metaphors (Mashal and Kasirer, 2011). In this study we used a multiple-choice task that included conventional metaphors (e.g., *sharp tongue*), novel metaphors (e.g., *joy bits*), and unrelated word-pairs (e.g., *laundry rabbit*). Unlike conventional metaphors, which are coded in the mental lexicon, novel metaphors require the computation of on-line interpretation. It might be the case that the ability to create novel semantic connections between apparently unrelated concepts is not impaired in ASD. Consistent with our results, Hermann et al. (2013) found that processing of novel metaphors in 20 adults with Asperger syndrome (aged 22–68) was similar to performance of age-matched TD individuals. The experiment included a semantic judgment task, in which participants were asked to make rapid decisions about the literal truth of a given sentence. The task consisted of four types of sentences: literally true high-typical sentences (e.g., *Some lawyers are juvenile magistrates*); literally true low-typical sentences (e.g., *Some lawyers are tax advisors*); metaphors (e.g., *Some lawyers are scale rulers*); and scrambled metaphors (e.g., *Some lawyers are days off spring*). Responses to metaphors were significantly slower than were responses to the other types of expressions, but this pattern of results characterized both groups, with no impairment in performance seen in the ASD group. Thus, there is evidence that people with ASD demonstrate no difficulties with figurative language, contrary to previous arguments in the literature.

Note that some earlier work has suggested that individuals with ASD do not comprehend figurative language due to difficulties in executive functions (Russell, 1997). According to this account, ASD impairs planning, set-shifting (flexibility), working memory, and inhibition (Ozonoff and Strayer, 1997). It has been suggested that executive dysfunction contributes to the typical communication and social impairments in ASD (Landa and Goldberg, 2005). However, evidence for executive dysfunction in ASD remains equivocal. For example, Robinson et al. (2009) found differences in inhibition, planning, and self-monitoring between individuals with ASD and TD participants, but no group differences in cognitive flexibility and verbal fluency were noted. Other studies have recorded difficulties in linguistic and categorical (semantic) fluency in ASD (Rumsey and Hamburger, 1988). Executive dysfunction has a considerable adverse impact on the ability to plan well formed novel linguistic constructs, and to shift from one meaning of a word or phrase to another, especially in the case of metaphoric language (Landa and Goldberg, 2005). The ability to combine the two words that form a metaphor to a meaningful expression might thus be more affected by deficits in executive functions than the ability to interpret literal expressions (Amanzio et al., 2008). Mashal and Kasirer () have indeed demonstrated that executive dysfunction contributed to comprehension of novel metaphors among children with ASD.

Executive functions are important not only for comprehension but also for the generation of metaphors (Dietrich, 2004; Chiappe and Chiappe, 2007). To generate a metaphor that contains a topic and a vehicle, a speaker must first select the property that will be attributed to the topic, and then scan semantic knowledge for suitable vehicles that exemplify the abstract, higher-order attributive category. This process requires that one maintains access to the category while inhibiting features of both topic and possible vehicles that are irrelevant to the higher-order category. That is, highly accessible but irrelevant semantic knowledge (e.g., adjectival descriptions of the topic) as well as many accessible but trite possibilities, such as idioms, clichés, and conventional metaphors must all be inhibited. Finally, the speaker must evaluate whether the metaphor conveys the desired meaning and emotional tone, and then revise it if necessary (Silvia and Beaty, 2012).

There is evidence that the generation of conventional and novel metaphors relies on different cognitive abilities (Bowdle and Gentner, 2005; Mashal, 2013). Silvia and Beaty (2013) suggested that generation of conventional metaphors is influenced by general knowledge or vocabulary, whereas generation of novel metaphors is affected by fluid intelligence and executive processes. Silvia and Beaty (2012) gave their participants two different prompts and instructed them to describe past emotional experiences through a metaphor. The first prompt asked people to “think of the most boring high-school or college class that you have ever had. What was it like to sit through?” For the next prompt, participants were asked to “think about the most disgusting thing you ever ate or drank. What was it like to eat or drink it?” Six measures of inductive reasoning, primarily non-verbal and visuo-spatial, were used to assess fluid intelligence. Results showed that fluid intelligence predicted the creative quality of novel metaphors. This suggests that people with high non-verbal intelligence can produce more creative metaphors than do people with lower intelligence.

Within the ASD literature, creativity has been measured primarily by non-verbal tasks that involved the production of spontaneous sequences of colors, tones, or drawings (Frith, 1972; Lewis and Boucher, 1991; Liu et al., 2011) or by verbal fluency tasks (Boucher, 1988). No extensive investigation of generation of novel verbal concepts has been conducted. Craig and Baron-Cohen (1999) examined creative thinking in children with ASD by using the Torrance Creativity Test (Torrance, 1974). Their participants could generate novel uses to an object (e.g., “what else can a pencil be?”), but they generated fewer such novel uses overall compared to TD children. Moreover, their ideas tended to be quite concrete rather than imaginary. Liu et al. (2011) emphasize that imagination is not necessarily important for successful performance on tasks that involve creativity and originality. They used an exercise in divergent thinking which included 12 incomplete figures, and asked their participants to complete the figures in an original way as well as to title them. The completed drawings were assessed on domains of fluency, openness, flexibility, originality, and elaboration. According to Liu et al., children with Asperger’s syndrome demonstrated better elaboration and originality than did age-matched TD peers.

The main goal of the current study is to examine metaphor comprehension and generation in adults with high-functioning ASD. Generation of novel and original metaphors is assumed to measure verbal creativity. In addition, we examine executive functions and analyze the contribution of these functions to performance on the metaphor tasks. We hypothesize that adults with ASD will show difficulty in comprehension of conventional metaphors but not in comprehension of novel metaphors. We further expect participants with ASD to be able to generate novel metaphors but to a lesser extent than TD participants. Finally, we assume that executive functions will contribute to metaphor comprehension and generation in both the ASD and the TD groups.

## MATERIALS AND METHODS

### PARTICIPANTS

Thirty-four participants took part in the study, 17 adults with high functioning ASD and 17 age-matched typically developing adults. The ASD group consisted of 14 men and 3 women, ranging in age from 18 to 27 (mean age = 21.06, SD = 3.44), and the TD group consisted of eight men and nine women, ranging in age from 18 to 25 (mean age = 22.71, SD = 2.02). All participants were native Hebrew speakers who completed at least 12 years of formal education, without learning disabilities or attention problems. The ASD group was recruited through classes integrated within regular high schools and through community services for persons with ASD. Diagnosis was done by a psychiatrist, in line with the Diagnostic and Statistical Manual of Mental Disorders-IV (American Psychiatric Association, 2013). Participants also completed the autism-spectrum quotient (AQ) questionnaire (Baron-Cohen et al., 2001), scoring above 26 on this measure (*M* = 29.17, SD = 2.03), as clinically accepted in Israel (Golan et al., 2009; Gold et al., 2010).

The TD group included 17 volunteers that were recruited from two different regular high schools and one college from central Israel. To be included in the study all participants had to score within the normal age-appropriate range on the screening tests described below. If a participant scored outside this range he or she did not continue with the experimental tasks. All participants signed an informed consent form prior to the research session.

### MATERIALS

#### Screening tests

All participants underwent screening tests that included the test of non-verbal intelligence (TONI-3; Brown et al., 1997), a Hebrew naming test (Kavé, 2005a), and the vocabulary sub-test from the Wechsler Adult Intelligence Scale (Wechsler, 1997). As can be seen in **Table 1**, the groups did not differ in TONI or naming, but the ASD group performed less well on the vocabulary measure.

**Table 1:** Mean correct responses (and SD) on screening tests, by group.

	ASD (*N* = 17)		TD (*N* = 17)		
	Mean	SD	Mean	SD	*t*
TONI-3	38.8	4.13	40.71	1.89	1.652
Hebrew naming	46.47	1.73	46.71	1.82	0.385
Vocabulary	48.18	6.46	55.82	3.34	4.334**

**p < 0.001.

Note: TONI-3, Brown et al. (1997), Hebrew naming test, Kavé (2005a), Vocabulary, Wechsler (1997).

#### Metaphor comprehension

We used the questionnaire developed by Mashal and Kasirer (2011) to test comprehension of conventional metaphors (e.g., *sharp tongue*) and novel metaphors (e.g., *pure hand*). For each metaphoric expression, four alternative responses were offered: a correct metaphoric interpretation, a literal interpretation, an unrelated interpretation, and a choice saying “this expression is meaningless.” Participants were instructed to choose the one answer they thought was the best of the four alternatives. The questionnaire consisted of 20 items and scores were the sum of all correct answers.

#### Metaphor generation

To examine generation of metaphors we constructed a questionnaire that contained 10 concepts relating to common emotions (e.g., *feeling sad*). Participants were asked to create and write down a new way of expressing the meaning of the concept. Instructions emphasized originality so as to encourage participants to create a new expression rather than simply paraphrase the one presented in the questionnaire. Thus, participants were asked “to create and write down a new expression, which is more comprehensible within your peer group than outside it.” We selected the concepts from the stimuli used by Levorato and Cacciari (2002) in their study of linguistic creativity.

A preliminary test was conducted with 20 Hebrew speaking adults (age range 18–25) who were presented with concepts and asked to generate new expressions. Eight concepts were presented in the form of a metaphor (e.g., *love is*____) and eight concepts were presented in the form of a simile (e.g., *feeling worthless is like*____). The aim of this procedure was to adjust the test to Hebrew. Three metaphors and three similes did not produce metaphoric responses and were thus excluded from the study. The final questionnaire included five metaphors and five similes.

Two judges independently coded the obtained responses. They were asked to decide whether each expression was literal or figurative. A novel metaphoric response (e.g., *feeling worthless is like a mirror smashed to pieces*) received three points, a conventional metaphor or idiom (e.g., *feeling embarrassed is like having a red face*) received two points, and literal responses or paraphrases (e.g., *feeling successful is like a victory*) received 1 point. Unrelated expressions received no points. The maximum score was 15 for metaphors and 15 for similes. Scores were converted to percentages.

#### Creativity

Creative ability was measured by the percent of responses that received three points on the generation test out of the 10 items.

#### Executive functions

Executive functions were assessed by the Ambiguous Word Meaning Generation Test (Mashal and Kasirer, 2011), verbal fluency tests (Kavé, 2005b), and the Trail Making Test (Reitan and Davison, 1974).

***Ambiguous Word Meaning Generation Test.*** This test investigates the ability to activate different meanings of ambiguous words and then shift between them (Mashal and Kasirer, 2011). Participants were presented with a list of 20 short unbiased sentences that ended with ambiguous words (e.g., *Look at this bank*). They were instructed to say aloud all meanings of the final word. For each participant, we counted the number of correct answers.

***Phonemic fluency.*** Following Kavé (2005b), phonemic fluency was assessed by obtaining the number of words generated in 1 min for the letters bet (b), gimel (g), and shin (sh). We used a sum score of all words produced on the three letters.

***Semantic fluency.*** Following Kavé (2005b), semantic fluency was assessed by obtaining the number of words generated in 1 min for each of the following three semantic categories: animals, fruits and vegetables, and vehicles. We used a sum score of all words produced on the three categories.

***The Trail Making test.*** This test consists of two parts, A and B, requiring participants to connect a series of digits placed in random order on a sheet of paper in ascending order (TMT A), and to connect a series of numbers and letters in ascending order while alternating between numbers and letters (TMT B) (Reitan and Davison, 1974). Scores were based on time to complete each task.

### PROCEDURE

Participants were tested individually in schools or in their homes. Screening tests were administered on a separate initial session. On a second session, half of the participants were tested on the executive functioning tasks and then on the metaphor comprehension and generation tasks. The rest of the participants were tested in the reverse order.

## RESULTS

### METAPHOR COMPREHENSION

**FIGURE 1:**
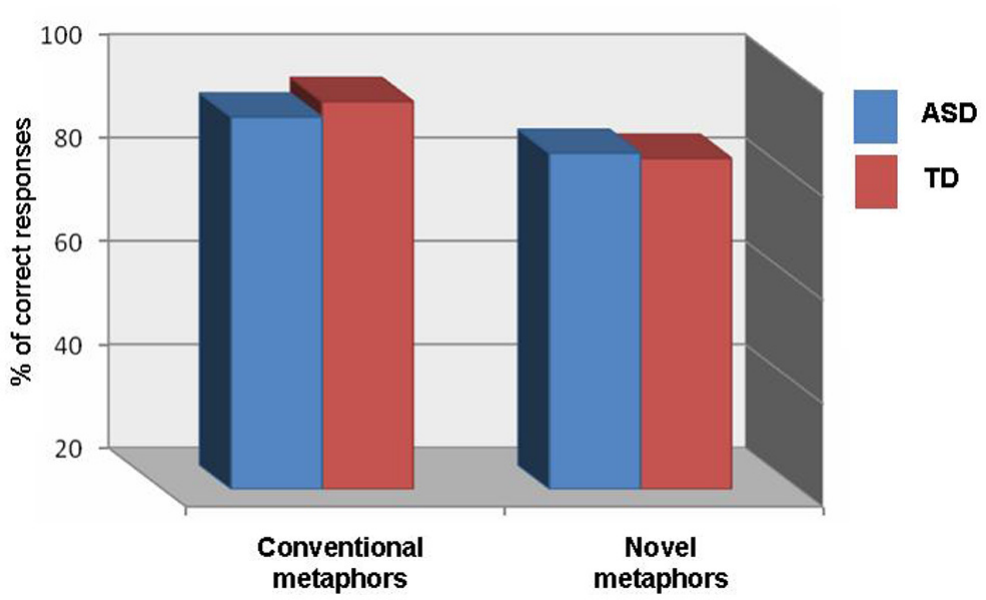
**Percent of correct responses on the metaphor comprehension questionnaire, by group**.

To examine performance on the comprehension questionnaire, a 2 × 2 repeated measures ANOVA was conducted, with group (ASD, TD) as the between-subject variable and type of metaphor expression (conventional, novel) as the within-subject variable. No main effect of group was found, *F*(1,32) = 0.979, *ns*, η^2^ = 0.030 (see **Table 2**). The main effect of metaphor expression was significant, *F*(1,32) = 5.725, *p* < 0.05, η^2^ = 0.152, with better performance on conventional metaphors (*M* = 93.53%, SD = 11.25) relative to novel metaphors (*M* = 84.12%, SD = 20.91). There was no significant interaction between group and type of expression, *F*(1,32) = 1.811, *ns*, η^2^ (see **Figure 1**).

**Table 2:** Mean correct responses (and SD) on the comprehension questionnaire, by type of metaphor expression and group.

	TD		ASD	
	Mean	SD	Mean	SD
Conventional	88.82%	14.09	98.24%	3.93
Novel	84.71%	18.41	83.53%	23.70

### EFFECTS OF VOCABULARY ON COMPREHENSION TEST SCORES

Since vocabulary scores were significantly lower in the ASD group than in the TD group, we examined the effects of vocabulary knowledge on performance. Vocabulary scores were significantly and positively correlated with comprehension of conventional metaphors, *r*(32) = 0.587, *p* < 0.001. There was no significant correlation between vocabulary scores and comprehension of novel metaphors, or between vocabulary scores and generation of either simile or metaphors. Because vocabulary scores were found to be significantly correlated only with comprehension of conventional metaphors, we used vocabulary as a covariate in a further analysis of group effects on this measure. When vocabulary was held constant, the effect of group remained non-significant, *F*(1,31) = 0.35, *p* = 0.56, η^2^ = 0.01.

### METAPHOR GENERATION

**FIGURE 2:**
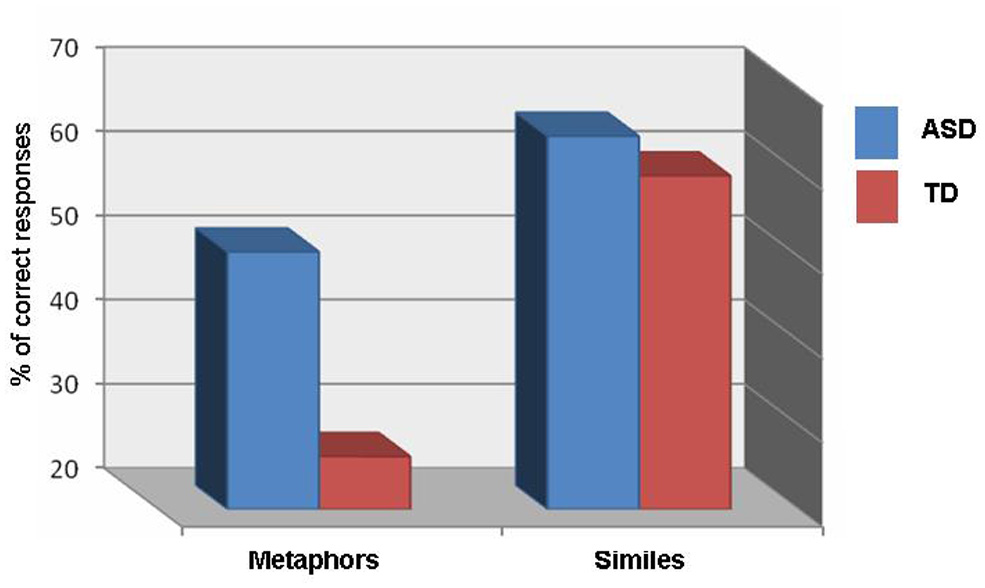
**Percent of correct responses on the generation questionnaire**.

To examine performance on the generation questionnaire, a 2 × 2 repeated measures ANOVA was conducted, with group (ASD, TD) as the between-subject factor and linguistic constraint (metaphor, simile) as the within-subject variable. No main effect of group was found, *F*(1,32) = 3.633, *ns*, η^2^ = 0.102 (see **Table 3**). The main effect of linguistic constraint was significant, *F*(1,32) = 30.460, *p* < 0.001, η^2^ = 0.488, with worse performance on metaphors (*M* = 38.43%, SD = 28.00) than on similes (*M* = 61.96%, SD = 25.08). There was also a significant interaction between group and, *F*(1,32) = 5.288, *p* < 0.05, η^2^= 0.142. A *post hoc* analysis revealed that the source of the interaction was in greater metaphor generation in the ASD relative to the TD group (*p* < 0.05). No significant group difference was found for simile generation (*p* = 0.59) (see **Figure 2**).

**Table 3:** Mean correct responses (and SD) on the generation questionnaire, by stimuli and group.

	ASD		TD		
	Mean	SD	Mean	SD	
Metaphor	50.59%	31.89	26.27%	16.91
Simile	64.31%	27.58	59.61%	22.91

### CREATIVITY

**FIGURE 3:**
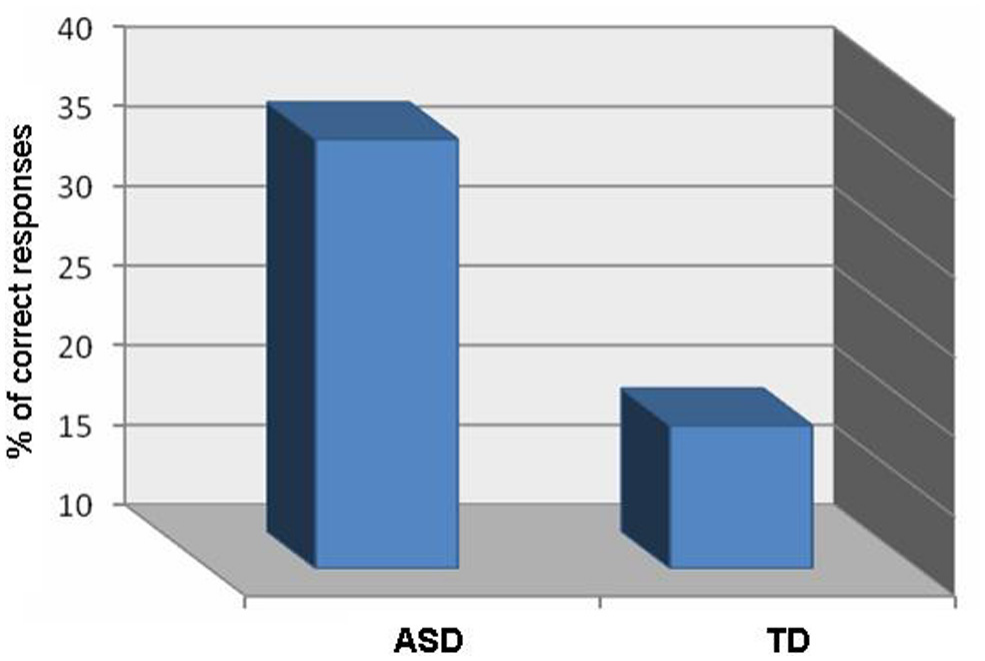
**Percent of original responses on the generation questionnaire, by group**.

A *t*-test for independent samples revealed a significantly greater percent of original responses (receiving a score of 3) within the ASD group (*M* = 37.06%, SD = 31.38) than within the TD group (*M* = 19.41%, SD = 11.97), *t*(32) = -2.166, *p* < 0.05 (see **Figure 3**).

Next we compared group performance on the five measures of executive functions with independent-samples *t*-tests. These analyses revealed significant group differences on all measures, with worse performance in the ASD group (see **Table 4**).

**Table 4:** Mean correct responses (and SD) on measures of executive functions, by group.

	ASD		TD		
	Mean	SD	Mean	SD	*t*
AMGT	16.88	4.04	19.47	0.80	2.588*
Phonemic fluency	28.47	11.11	39.47	9.07	3.162**
Semantic fluency	44.71	12.59	57.53	8.88	3.429**
TMT-A	43.88	18.48	30.06	9.02	-2.771**
TMT-B	76.94	30.96	59.00	16.01	-2.122*

*p < 0.05, **p < 0.01.

*Note*: AMGT Ambiguous Words Meaning Generation Test; TMT Trail Making.

### PREDICTION OF METAPHOR SCORES BY BACKGROUND TEST SCORES

To examine which test predicts comprehension and generation of metaphors, we used a set of four hierarchical and stepwise regression analyses. Predictors included scores on the screening tests (TONI, naming, and vocabulary) as well as scores on the tests of executive functions (AMGT, fluency, and TMT). For the comprehension test, we conducted one analysis with conventional metaphors as the predicted variable and one analysis with novel metaphors as the predicted variable. Similarly, for the generation task we conducted one analysis for metaphors and one analysis for similes. In each regression we first entered the group variable (ASD, TD), and then we entered scores on the three screening tests and the five measures of executive functioning in a stepwise manner. Results of these regression analyses are presented in **Table 5**. The order of the variables presents the order of significance. As can be seen in the table, performance on both the screening tests and the tests of executive functions explained a significant share of the variance across study groups in all four analyses but each dependent variable was predicted by different scores.

**Table 5:** Summary of regression analyses predicting comprehension and generation by screening tests and measures of executive functions across groups.

		Predictor variable	*B*	SE B	β	*R*^2^	Δ*R*^2^	*t*
Comprehension	Conventional metaphors	Group	-2.98	3.84	-0.13	0.18*	0.18*	-0.77
		Vocabulary	0.78	0.32	0.44	0.35***	0.17**	2.51*
		Naming	1.90	0.90	0.30	0.43***	0.08*	2.11*
	Novel metaphors	Group	8.39	6.98	0.20	0.00	0.00	1.20
		TMT-B	-0.40	0.14	-0.50	0.22*	22.0**	-2.94**
Generation	Metaphor	Group	30.00	8.52	0.54	0.19**	0.19**	3.52***
		TONI-3	3.12	1.31	0.37	0.32**	0.13*	2.38*
	Simile	Group	12.41	7.77	0.25	0.01	0.01	1.60
		TONI-3	4.23	1.19	0.56	0.29**	0.28***	3.54***

*p < 0.05, **p < 0.01, ***p < 0.001.

Comprehension of conventional metaphors was best predicted by vocabulary and picture naming, *F*(3,30) = 7.71, *p* < 0.001, which accounted for 43.5% of the variance. Comprehension of novel metaphors was best predicted by TMT-B, *F*(2,31) = 4.34, *p* < 0.05, which accounted for 21.9% of the variance. Generation of metaphors was best predicted by TONI-3, *F*(2,31) = 7.25, *p* < 0.01, which accounted for 31.9% of the variance, and so was the generation of simile, *F*(2,31) = 6.46, *p* < 0.01, in which the TONI-3 score accounted for 29.4% of the variance. Group differences predicted metaphor generation with a positive beta coefficient, indicating that participants with ASD had higher scores on this test than did TD participants.

## DISCUSSION

The present study examined metaphor processing in adults with ASD, differentiating between conventional and novel metaphors on tests of comprehension and generation. Our results show that adults with ASD demonstrate no difficulties in comprehension of conventional and novel metaphors. Furthermore, adults with ASD outperformed age-matched TD peers in metaphor generation. An inspection of the type of metaphors generated indicated that the ASD group produced more original and creative metaphors than did the TD group.

Whereas previous studies highlighted difficulties with metaphoric language comprehension in ASD (e.g., Happé, 1993, 1995; Ozonoff and Miller, 1996; Rundblad and Annaz, 2010; Kaland et al., 2011; Mashal and Kasirer, 2011), the current research shows similar comprehension of metaphors among ASD and age-matched TD peers. One of the differences between our study and earlier studies might be that we examined adults, unlike previous studies that focused primarily on children and adolescents. It is possible that the accumulated verbal knowledge that comes with age leads to greater familiarity with conventional metaphors within the ASD group. Indeed, previous studies have emphasized the contribution of vocabulary to the comprehension of metaphors (Evans and Gamble, 1988; Nippold, 1998; Chiappe and Chiappe, 2007; Silvia and Beaty, 2013). Consistent with this observation, we found that higher vocabulary scores were correlated with better comprehension of conventional metaphors (but not with comprehension of novel metaphors). The fact that we found equivalent comprehension across groups might also be related to the task that we used. It is possible that presenting metaphors in a multiple-choice questionnaire facilitated comprehension and was easier than were previously used tasks, such as asking comprehension questions about metaphoric stories (Rundblad and Annaz, 2010), requiring sentence completion (Norbury, 2005), or having participants perform a semantic judgment task (Hermann et al., 2013).

As expected, and in line with previous studies (Mashal and Kasirer, 2011; Melogno et al., 2012; Hermann et al., 2013), no difference was found between participants with ASD and TD age-matched peers in comprehension of novel metaphors. Novel metaphor interpretation is not coded in the mental lexicon and hence is not dependent on previous knowledge. Indeed, unlike the correlation between vocabulary and comprehension of conventional metaphors, no association was found between vocabulary and comprehension of novel metaphors. The ability to understand novel semantic connections between seemingly unrelated concepts appears to be intact in ASD, probably because it does not rely on activation of lexicalized expressions. Consistent with this finding, Melogno et al. (2012) emphasized that individuals with ASD can explain novel metaphoric phrases in unique ways that rely on phonological or semantic association. Thus, comprehension of novel metaphor is intact in ASD because it relies on good associative abilities rather than on lexicalized verbal knowledge.

Another finding of the current study is that contrary to our hypothesis, participants with ASD generated more metaphors than did age-matched peers. We were specifically interested in testing whether adults with ASD relied on previous knowledge, and were thus using familiar metaphors or idioms, or alternatively, whether they generated their own novel and original metaphors. We found that adults with ASD demonstrated greater verbal creativity than did TD individuals. Examples of creative sentence completions included phrases such as “Feeling successful is like seeing the view from the mountaintop” and “Feeling worthless is like offering a salad to South Americans.” These examples contrasted with more conventional figurative expressions provided by TD adults, such as “Feeling sad is to get the blues.” Our results suggest that adults with ASD can create unique verbal associations that are not restricted to previous knowledge, thus pointing to unique verbal creativity in ASD. It has been reported that one in ten people with autism shows some savant skills in such categories as music, art, calendar calculations, or mathematics (Treffert, 2009). Yet, most studies have indicated impoverished creativity in autism. For example, in standardized tests such as the Torrance Creativity Test (Torrance, 1974), people with ASD demonstrate difficulties in cognitive flexibility and imaginative fluency, as well as lack of imagination and originality compared to TD participants (Craig and Baron-Cohen, 1999).

Why, then, do adults with ASD demonstrate greater creativity in metaphor generation when previous studies found that they were lacking in imagination? Baron-Cohen et al. (2009) suggest that the hypersensitivity that characterizes autism gives rise to excellent attention to details. With respect to verbal creativity, this attention to details is associated with weak central coherence, leading to greater appreciation of local features over global ones. Lyons and Fitzgerald (2005) emphasized that in contrast to their social impairments individuals with Asperger syndrome are gifted with creativity and originality, excellent memory, strong focus of attention and specific cognitive styles. The unique verbal associations generated by the ASD group in the current study could thus reflect memory for details and weak central coherence. Another reason why adults with ASD generated more original metaphors might relate to difficulty in theory of mind. Mind-blindness makes one focus on one’s own thoughts, ignoring the addressee (Happé and Vital, 2009), possibly leading to production of expressions that are less conventional (Liu et al., 2011). Kanner (1946) was the first to note that the unique phrases produced by the children he studied could be interpreted as metaphoric language. Asperger (1941) also identified certain expressions in the speech of his patients that resembled the novel linguistic forms produced by young TD children. Asperger proposed that characteristics such as concrete intelligence and disregard of social conventions might be prerequisites for certain forms of new thinking and creativity (Gillberg, 2002). Fitzgerald (2004) also noted that individuals with Asperger syndrome have remarkable capacities for persistence and observation, high levels of energy and motivation, and abilities to focus intensely on a single topic. It appears that some individuals with autism and Asperger syndrome are highly creative, imaginative and original and even their humor can range from word-play and sound associations to precisely formulated, truly witty comments. According to Lyons and Fitzgerald (2004) some individuals with autism and Asperger syndrome also seem to master the cognitive processing of humor, i.e., incongruity and its resolution and switching of meanings as portrayed by the production of relatively sophisticated puns and word games.

In our final analysis we sought to identify the extent to which executive functions as well as verbal and non-verbal skills contribute to the prediction of metaphor comprehension and generation. We found that while verbal abilities, i.e., vocabulary and picture naming, contribute to the comprehension of conventional metaphors, this is not the case with regard to novel metaphors, in line with previous studies (e.g., Nippold, 1998; Chiappe and Chiappe, 2007; Silvia and Beaty, 2013). Furthermore, executive functions were shown to predict novel metaphor comprehension, as has been previously reported (Pennington and Ozonoff, 1996; Amanzio et al., 2008; Mashal and Kasirer, 2011; Mashal, 2013). More specifically, the TMT-B, which demands mental flexibility, contributed to the prediction of novel metaphor comprehension. We believe that comprehension of novel metaphors relies on flexibility because it is based on a shift between the literal and the metaphoric meanings of the words that appear in the new expression. Champagne-Lavau and Stip (2010) also found this correlation between the TMT-B and metaphor comprehension in schizophrenic and TD adults.

Finally, metaphor generation was predicted by non-verbal cognitive ability. Indeed, Silvia and Beaty (2012) have also emphasized the contribution of fluid intelligence to the generation of creative metaphors. It is possible that generation of metaphors, specifically novel ones, relies on the ability to come up with new solutions, assessed by tests of fluid intelligence. We note that a similar connection was documented in our study between non-verbal intelligence and simile generation, although no group differences in simile generation were found.

Some limitations of the study should be taken into account. Our measures of executive functions were rather limited and may need to be expanded to include the assessment of working memory and inhibition. Studies have shown that people with ASD may present difficulties in tasks that require response inhibition (e.g., Hughes and Russell, 1993; Hughes, 1996; Russell, 1997; Minshew et al., 1999; Hill, 2004; Robinson et al., 2009) or when they are required to shift from one response set to another (Ozonoff and Strayer, 1997; Ozonoff et al., 1998). It is possible that low response inhibition led to generation of unique verbal expressions by our ASD participants. Future studies will have to examine whether inhibition is associated with metaphor generation in ASD.

In sum, our findings indicate that adults with ASD are not impaired on comprehension of conventional and novel metaphors. We also found that adults with ASD generate more original metaphors relative to age-matched peers and that non-verbal skills contribute to this ability. The study points to unique verbal creativity in ASD which has not been studied in this way before.

## Conflict of Interest Statement

The authors declare that the research was conducted in the absence of any commercial or financial relationships that could be construed as a potential conflict of interest.
